# Supplementary material for: Are carbon emissions trading and green financial instruments synergistic? -Comprehensive quantitative research based on content analysis
Source: PLoS One. 2024 Mar 7;19(3):e0298601. doi: 10.1371/journal.pone.0298601 (PMC10919601; doi:10.1371/journal.pone.0298601)
Supplement: S1 Data — (ZIP) [file pone.0298601.s001.zip › Data/地级市Z_F工作报告.zip!/║╙─╧/╨┼╤⌠╩╨/└┤╫╘╣1⁄2╓┌║┼í╛┬φ┐╦╩2╛▌═°í┐_2014.docx]

各位代表：

　　现在，我代表信阳市人民政府，向大会报告工作，请予审议，并请各位政协委员和其他列席的同志提出意见。

　　一、2013年工作回顾

　　过去的一年，面对极其错综复杂的形势和艰巨繁重的任务，在省委、省政府和市委的正确领导下，全市上下认真贯彻落实党的十八大和十八届二中、三中全会精神，抢抓中原经济区建设和产业转移的重大机遇，发挥优势，持续求进，务实发展，积极作为，全市经济社会发展持续保持了好的趋势、好的态势、好的气势。

　　一年来，我们主要抓了以下工作：

　　（一）坚持促增长调结构，经济增长质量和效益进一步提高。面对去年我市第一季度主要经济指标增速回落较快、促增长压力加大的严峻形势，我们把促增长调结构作为经济工作的首要任务，科学应对和有效解决经济运行中的突出矛盾和问题，经济增长的速度、结构、质量、效益在持续求进中趋于协调。发展态势在“进”中向好。加强经济运行调节，及时采取一系列促增长措施，大力实施重点项目攻坚计划和工业促增长调结构攻坚行动，加强产销对接、用工对接、银企对接，加快122个市重点项目和156个重大工业结构调整项目建设，扩大投资和消费需求，优化发展环境，激发市场主体活力，有效扭转了经济下行趋势，经济增长从第二季度实现逐季回升。初步核算，全市生产总值增长9.1%，规模以上工业增加值增长14.7%，固定资产投资增长23%，社会消费品零售总额增长13.9%。产业结构在“进”中优化。强化新型农业现代化基础，大力推进粮食生产核心区建设、高标准粮田“百千万”建设工程、现代农业产业化集群培育工程和主食产业化工程，新增高标准粮田76.2万亩，省级示范性现代农业产业化集群达到16个。粮食生产克服连续干旱，总产达到117.14亿斤，实现了“十连增”；坚持新型工业化主导，全市工业投资完成557亿元，增长28.4%，增速居全省第3位，对固定资产投资的贡献率达44.8%；加快现代服务业发展，第三产业增加值突破500亿元，增长9.3%，增速居全省第4位。物流快递、信息服务、电子商务、金融保险、文化创意等现代服务业加快发展，义乌国际商贸城等一批商贸物流项目落户信阳。我市被确定为全省集中连片推进农产品流通和农村市场体系建设试点市。旅游业健康发展，全年接待旅游者1800万人次，旅游综合收入95.7亿元。质量效益在“进”中提升。全市财政总收入93.1亿元，增长20.3%。其中，地方公共财政预算收入67.9亿元，增长22.5%；税收完成51.7亿元，占地方公共财政预算收入的76.1%，高出全省平均水平3.1个百分点。预计全市规模以上工业实现主营业务收入1680亿元、利税142亿元、利润90亿元，分别增长17.3%、10.7%和16.2%。

　　（二）坚持强载体建体系，发展科学性和协调性进一步增强。突出抓好“一个载体、三个体系”，着力增强经济发展的科学性、协调性和可持续性。加快产业集聚区建设。按照“四集一转”要求，围绕“两提两扩两优”目标，突出主导产业集群发展，强化政策扶持和项目支撑，强力推进重点项目攻坚督导和联合督查，引龙头、抓延链、建基地、育集群，我市高端制造、新型建材、绿色食品三大产业基地和电子信息、现代家居、纺织服装、现代物流等主导产业初具规模，集群集聚态势正在形成。预计全市产业集聚区完成固定资产投资799.2亿元，增长31.4%，占全市固定资产投资的比重达到54.3%；预计产业集聚区规模以上工业主营业务收入1128亿元，增长21.3%，占全市规模以上工业主营业务收入的67%，产业集聚区的综合带动作用日益增强。推进新型城镇化健康发展。启动了信阳市城市总体规划（2013-2030）修编工作，完成了总规纲要编制任务。统筹推进中心城市、县城和中心镇协调发展，新区开发、老城改造和中心城市组团建设全面加快，商务中心区、特色商业区的规划建设有序推进，浉河三期治理和中心城区内河治理、雨污分流、供水管网等重点工程进展顺利，市污水处理二期工程通水运营，新七大道顺利贯通。中心城区建设完成投资344亿元。持续深化拓展“六城联创”，城市精细化、数字化管理水平进一步提升，中心城市的辐射带动能力和城镇载体功能日益增强。全市城镇化率提高到近40%，城乡一体化发展步伐进一步加快。我市成功再创全省文明城市，荣获中国最美丽城市、中国最安全城市称号，连续五年蝉联中国十佳宜居城市、连续三年蝉联中国最具幸福感城市。积极构建自主创新体系。出台了关于创新驱动发展的若干意见，实施了一批重大科技专项。全市新增院士工作站1家、省级工程技术研究中心5家，设立博士后科研工作站2家、博士后研发基地5家，争取省级高新技术产业化项目2个。我市企业牵头成立的“稻米精深加工产业技术创新战略联盟”首次列入国家级试点，农科院培育的2个水稻新品种通过省级审定。全市高技术产业增加值增长32.5%，高于全市规模以上工业企业增加值增速17.8个百分点，科技进步对经济增长的贡献率进一步提高。

　　（三）坚持打基础创优势，发展支撑和后劲进一步提升。围绕重点领域和关键环节，着力打基础增后劲，不断巩固、放大和提升发展优势。强化提升前沿优势。突出“五网一系统”建设，境内312和107国道改建工程、宁西铁路复线电气化改造和京港澳高速公路驻信段改扩建工程加快推进，淮河航运基础设施进一步完善；智慧信阳、3G网络、信阳呼叫中心等重大项目进展顺利。放大提升生态优势。大力创建国家卫生城市和全国绿化模范城市，实施林业生态市建设提升工程，加强淮河源国家重点生态功能区保护和南湾湖国家重点湖泊生态保护，完成大唐华豫电厂1号机组脱硝工程。我市单位生产总值能耗、二氧化碳排放和主要污染物减排总量均可完成省下达目标，大气和水环境质量稳居全省第1位。巩固提升民智优势。继续实施全民技能振兴工程和“以职教促就业”计划，加强职教实训基地建设，搭建农民工技能培训、就业与企业用工对接平台。全年完成各类职业技能培训21.8万人次，产业集聚区新增就业5.5万人。大力实施“回归创业工程”，全市新增返乡创业企业1056家。

　　（四）坚持抓改革促开放，发展动力和活力进一步释放。深入推进重点领域改革。农村改革发展综合试验区建设向纵深推进，土地流转、金融创新、专业合作、社会保障、城乡统筹等项改革取得新进展。扎实推进行政审批制度改革，新取消和下放市级审批事项67项、合并6项。积极推进公共资源交易市场化改革，探索网上招投标新方式。深化国有企业改革，国有粮食企业产权制度改革有序推进，市直机关所属企业脱钩改制工作取得积极进展。大力实施促进民营企业中小企业健康发展行动计划，非公经济总量占生产总值的比重达56.7%，较上年提高1个百分点；民间投资完成1229亿元，占全市固定资产投资的比重达83.5%。积极推进政府机构改革和事业单位分类改革，固始县实行了省直管县体制。财税金融改革不断深化，“营改增”试点有序展开，农村信用社改革加快推进，村镇银行经营规模持续扩大。医药卫生、文化教育、广播影视、社会保障、人口和计划生育等社会事业改革进一步深化。户籍、林权、水利、供销等项改革顺利实施。电力、天然气、成品油等资源性产品价格改革稳步推进。不断完善要素保障机制。全市金融机构各项存款余额1830.1亿元，较年初增加293.5亿元，增长19.1%，增幅居全省第2位。全市贷款余额909.5亿元，较年初增加167.3亿元，增长22.6%，增幅居全省第3位。新增存贷比达到57%，为近年来最高。全市资本市场融资69.9亿元，羚锐、华英两家上市公司首次实现再融资。民生银行、洛阳银行在信阳设立分支机构，填补了我市没有大型民营股份制银行的空白。充分运用城乡建设用地增减挂钩和人地挂钩试点政策，全年争取建设用地指标6.4万亩，土地保障能力进一步增强。集中开展中心城区“三违”整治工作，维护了良好的土地管理秩序。全面提升对外开放水平。全年实际利用外商直接投资4.2亿美元，增长20.8%；实际到位省外资金180亿元，增长20%；外贸出口总值2.9亿美元，增长13.2%。全面推进战略合作，成功举办第21届信阳国际茶文化节，与省直部门、央企和战略投资者签订了一批新的战略合作协议。持续推进“四一”招商行动，专业化集群化招商取得明显成效，又有一批龙头型、基地型项目落户信阳。

　　（五）坚持保底线惠民生，社会和谐稳定大局进一步巩固。牢牢把握发展、民生、安全、稳定底线，集中财力保障民生建设，省十项重点民生工程全面完成，市十件实事进展顺利。全市财政用于改善民生的支出229.6亿元，占财政总支出的75.5%。千方百计扩大就业。实施积极的就业扶持政策，支持创业带动就业，全市城镇新增就业10.7万人，下岗失业人员实现再就业3.6万人，就业困难人员实现就业1.2万人，均超额完成省定目标，城镇登记失业率控制在2.33%。城镇居民人均可支配收入19150元，农民人均纯收入7982元，分别实际增长8.4%和11.2%，增幅均居全省第2位。不断完善社会保障体系。社会保险全部实现市级统筹，养老保险和医疗保险实现全覆盖。城镇居民医保补助标准从每人每年240元提高到280元，新农合参合率达到99.44%，筹资水平从每人每年290元提高到340元，重大疾病保障病种从20个扩大到35个。企业离退休人员基本养老金、城乡低保、农村五保对象补助标准稳步提高，住房公积金制度覆盖面进一步扩大。保障性住房开工建设22121套，竣工14552套，超额完成年度目标任务。持续改善群众生活质量。城市新增管道天然气用户3.6万户，新改建乡村公路589公里，完成农村危房改造1.73万户，新解决72.25万农村居民及师生饮水安全问题。加大农田水利基本建设，新打机井2277眼，整修塘堰5916口，治理水土流失面积181平方公里，新增灌溉面积18.9万亩。加快畜禽、水产、蔬菜等无公害农产品基地建设，市民“菜蓝子”更加丰富。扎实推进美丽乡村建设，全市建成传统文化村98个，有4个村被确定为中国美丽乡村首批创建试点、7个村被列入中国传统村落名录、5个村列入河南省特色旅游村名录，平桥区郝堂村成为河南省唯一被命名的全国首批美丽宜居村庄。加大扶贫工作力度，新完成132个贫困村整村推进任务，又有12万农村贫困人口实现稳定脱贫。全面发展社会事业。大力实施“双千”工程，引进教育、卫生系统基层急需专业人才2052名。完成学前教育三年行动计划，改造农村义务教育薄弱学校28所，职业教育、普通高中教育、高等教育取得新的成果，信阳农业高等专科学校成功升格为信阳农林学院。城乡医疗和公共卫生服务体系不断完善，全市医院、卫生院新增床位2003张，政府举办的基层医疗卫生机构全部实施国家基本药物制度，12类基本公共卫生服务免费向城乡居民提供，重大传染病得到有效防控，艾滋病“四免一关怀”政策全面落实。大力实施广播电视村村通、农家书屋、农村电影放映、文化信息资源共享等文化惠民工程，人民群众的精神文化生活更加丰富。广泛开展全民健身运动，成功举办第五届全国自行车公开赛和我市第四届全民运动会。统筹人口与计划生育工作，人口自然增长率控制在5.1‰。慈善、社会福利、老龄、妇女儿童、残疾人等事业全面发展。积极创新社会治理。进一步完善信访“四家”工作法和“四有”工作机制，认真落实领导干部接访制度，集中处置一批信访积案，集中解决拆迁安置遗留问题，妥善化解了一些突出矛盾和问题。加强平安信阳建设，深化社会治安综合治理，完善公共安全应急管理体系，强化检验检疫、食品药品、安全生产和质量监管，严厉打击违法犯罪，社会和谐稳定局面进一步巩固，群众安全感进一步提升。

　　（六）坚持抓两转促两提，政府自身建设进一步加强。坚持依法行政，自觉接受人大法律监督和工作监督，主动接受政协民主监督和社会监督，认真办理人大代表建议和政协提案，深入推进“六五”普法和依法治理，严格规范公正文明执法，加快推行服务型行政执法，法治信阳建设取得积极进展。扎实推进“两转两提”，全面深化政务公开，强化行政监察、行政复议、行政执法监督和审计监督，加强反腐倡廉工作，政府自身建设迈出新的步伐。严格落实中央“八项规定”和省委省政府20条意见、市委市政府30条意见，严格执行党风廉政建设各项规定和我市“五禁止”、“十不准”规定，认真解决“四风”问题，突出治理庸懒散软，干部作风建设进一步加强，行政效能进一步提高，发展环境进一步优化。

　　一年来，我们加强社会主义核心价值观教育和精神文明建设，弘扬“大别山精神”，公民道德素质和社会文明程度明显提升。积极开展“双拥”共建活动，军政军民团结进一步加强，民兵预备役建设取得新进展。驻信解放军和武警、消防部队在支持地方建设、维护社会稳定等方面作出了重要贡献，工会、共青团、妇联和科协、文联、社科联、工商联、红十字会等人民团体在经济社会发展中发挥了不可替代的作用，民族、宗教、外事、侨务、对台工作全面加强，统计、物价、机关事务、地质、气象、地震、人防、农机、档案、史志等工作取得新成绩。

　　尤为可喜的是，一年来，我们坚持持续求进，实现了发展新突破。全市生产总值、全社会固定资产投资、规模以上工业主营业务收入在2010年突破1000亿元的基础上，再次突破1500亿元，综合实力迈上了新的台阶；各县区公共财政预算收入均突破2亿元，各县区全社会固定资产投资均突破100亿元，全市产业集聚区主营业务收入均突破50亿元，县域经济实现了新的发展；全市公共财政预算支出突破300亿元，其中民生支出突破200亿元，重点保障能力得到持续提升，信阳加快发展的基础更加坚实。

　　一年来，我们坚持务实发展，积蓄了发展新能量。持续关注工业、研究工业、学习工业、支持工业，全市规模以上工业企业达到1088家，新增122家，其中主营业务收入超过10亿元的企业22家，超过50亿元的企业2家；全市规模以上高成长性产业增加值突破300亿元，占全市规模以上工业增加值的71.9%；信阳电子信息、绿色食品、现代家居被列为省重点培育的千亿元产业集群。坚持项目带动，全市“十大项目”完成投资157亿元，建成投产重大工业结构调整项目64个，其中亿元以上项目33个。信阳电子信息产业集聚示范区加快发展，已集聚电子信息企业68家，建成投产17家，伯皇IT产业园、深圳手机产业园开工建设。信阳国际家居产业小镇建设加快推进，已完成基础设施投资11亿元，进场开工建设企业11家。“中国光彩事业信阳行”活动将于今年上半年正式启动，信阳加快发展的动力更加强劲。

　　一年来，我们坚持积极作为，创造了发展新优势。着力抓战略、谋长远，《大别山片区区域发展与扶贫攻坚规划(2011-2020年)》正式实施，《大别山革命老区振兴发展规划（草稿）》已由省发改委编制完成，《淮河生态经济带发展规划》进入国家战略研究层面；信阳高新技术产业开发区和潢川经济技术开发区创新升级工作得到省政府重视和支持，正在积极推进；出山店水库和明港机场前期工作取得重大进展，信阳海关即将通关运营，信阳东500千伏输变电工程加快推进，我市加快发展的综合优势更加明显。

　　各位代表！

　　在困难和挑战面前，我市取得这样的成绩确实不易。虽然经济增速低于预期目标，但仍处于合理区间，与全国、全省趋势一致，而且呈逐季回升态势，结构、质量、效益、就业等指标显著好于预期。这是省委、省政府和市委正确领导的结果，是人大、政协监督支持的结果，是全市人民共同奋斗的结果。在此，我代表市人民政府，向全市广大工人、农民、知识分子、干部职工，向驻信部队和武警官兵、公安民警以及社会各界人士致以崇高的敬意和衷心的感谢！

　　各位代表！

　　我们清醒地看到，我市前进道路上还面临很多矛盾和问题，政府工作还存在一些差距和不足：一是宏观环境错综复杂，经济运行中的不确定、不稳定因素依然较多，部分行业和企业生产经营还十分困难，我市经济基础薄弱、经济总量不高、人均水平较低的基本市情尚未根本改变，加快发展的任务依然艰巨；二是深层次矛盾依然突出，工业化、城镇化水平不高，城乡之间、县区之间发展还不均衡，三次产业、三大需求之间结构还不协调，开放的广度深度不够，发展优势还未充分转化为竞争优势；三是财政保障公共服务的能力仍然较弱，就业、教育、医疗、住房、食品药品安全等方面还存在薄弱环节，征地拆迁、涉法信访、安全生产等领域还存在群众反映强烈的问题，改善民生和加强社会管理的任务依然繁重；四是政府职能转变还不到位，一些公务人员的能力素质还不适应新形势新任务要求，一些领域的不正之风仍不同程度存在。对此，我们继续采取有效举措，认真加以解决。

　　二、2014年政府工作总体要求和主要目标

　　当前，我市面临着难得的发展机遇：从全球看，主要经济体经济持续复苏的趋势没有改变，国际经济大环境总体会比去年有所改善，世界经济仍将延续缓慢复苏态势。从全国看，中央确定今年为改革年，强调“稳中求进”是今年经济工作的总基调，全面深化改革必将进一步释放新的动力和活力。从全省看，我省深入实施三大战略规划，着力推进“一个载体、三个体系”建设，加快发展的外部环境不断优化、支撑能力不断增强。省委、省政府明确提出，今年经济工作的核心是“调中求进、变中取胜、转中促好、改中激活”，根本目的也是要通过更高质量的较快发展，打造河南经济升级版。从信阳看，我市正处于工业化、城镇化加快发展时期，面临着国家支持中原经济区、革命老区、贫困地区加快发展和承接产业转移的重大机遇，随着我市一系列打基础、增后劲、管长远的重要举措逐步见效，前沿、生态和民智优势形成的综合竞争力日益彰显，信阳已成为沿海地区产业向中部地区加速转移的“桥头堡”，发展前景良好。我们一定要坚定信心、抓住机遇、发挥优势、乘势而上，牢牢把握发展主动权。

　　今年政府工作的总体要求是：全面贯彻落实党的十八大和十八届二中、三中全会精神，认真落实省委、省政府和市委决策部署，紧紧围绕富民强市目标，抢抓中原经济区建设和产业转移机遇，充分发挥前沿、生态、民智优势，深入推进“一个载体、三个体系”建设，把改革创新、扩大开放贯穿于经济社会发展各个领域各个环节，切实提高经济发展质量和效益，促进信阳经济社会持续健康发展、社会和谐稳定。

　　今年全市经济社会发展的主要预期目标是：在优化结构、提高质量效益的基础上，生产总值增长9.5%以上，地方公共财政预算收入增长13%，全社会固定资产投资增长21%，社会消费品零售总额增长14%左右，外贸出口总额增长20%左右；城镇居民人均可支配收入、农民人均纯收入分别实际增长9%左右；城镇新增就业9万人左右；人口自然增长率控制在6.5‰以内；居民消费价格涨幅控制在3.5%以下；节能减排完成省下达任务。

　　今年经济增长预期目标定为9.5%以上，是根据我市发展需要和可能确定的，体现了中央和省里要求，也是在加快发展、扩大规模的同时，引导各方把主要精力和工作重心放到提质增效、转型升级上来，以实现更高水平、更好质量、更可持续的发展。

　　做好今年政府工作，必须把握“总要求”，继续坚持持续求进、务实发展、积极作为，努力做到调中求进、变中取胜、转中促好、改中激活。坚持“总布局”，把中国特色社会主义“五位一体”总体布局和我省打造“富强河南、文明河南、平安河南、美丽河南”，推进“两项建设”总体部署具体化，着力打造良好的自然生态、经济生态、社会生态、文化生态和政治生态。发挥“三优势”，抢抓中原经济区建设和产业转移的重要机遇，充分发挥我市前沿、生态和民智优势，深入推进“一个载体、三个体系”建设，全面加快信阳“一市一区两枢纽一基地”建设，着力培育竞争新优势、注入发展新活力、构建开放新格局。抓好“五关键”，围绕省委、省政府提出的“五着力”，重点把握好五个方面：一是必须以改革创新统领全局。坚持以十八届三中全会精神为指导，用改革的精神、改革的思路和改革的办法来破解发展难题，推动各项工作。二是必须以提质增效为中心任务。把提高经济社会发展质量和效益作为中心工作，更加注重抢抓机遇、加快发展、扩大总量，更加注重深化改革开放和强化创新驱动，更加注重扩大就业和改善民生，更加注重绿色循环低碳发展，着力在扩需求、转方式、调结构、促开放、惠民生上实现新突破。三是必须以人为核心推进新型城镇化。坚持以人为本、优化布局、生态文明、传承文化、产城互动、科学有序的基本原则，加快构建以人口城镇化为核心，以集约智能绿色低碳为特征，以科学规划为引领，以产业发展为支撑，以提高城镇综合承载能力为保障的新型城镇化格局。四是必须以放大提升和转化优势为抓手。立足信阳基础条件、所处发展阶段和外部环境，将我市前沿、生态、民智三大优势提高到战略层面深化研究，推动三大优势由局部突破向整体提升转变，转化战略优势，优化战略布局，强化战略支撑，推进战略合作，培育壮大战略支撑产业，努力做强工业、做大服务业、做优农业，不断增强综合竞争力，为经济社会发展提供持久支撑和保障。五是必须以保障和改善民生为目的。把保障和改善民生作为根本出发点和落脚点，加大民生投入，办好民生实事，不断促进公共资源配置均等化，让人民群众更多更公平享受公共服务；加强生态建设和环境保护，持续提高环境质量；加强社会建设，创新社会治理，增强社会发展活力，维护社会大局稳定。

　　各位代表！

　　加快发展是信阳解决所有问题的基础和关键。我们要扭住发展不放松，盯紧目标不动摇，团结拼搏不懈怠，脚踏实地，干在实处，当好前锋，努力完成全年经济社会发展各项目标。

　　三、2014年重点工作

　　今年，着重做好九个方面的工作：

　　（一）加快产业集聚区建设，着力在做强新型工业上下功夫。以加快建设中部地区承接产业转移的重要基地为目标，大力实施“四大工程”、“三大计划”，进一步做大做强产业集聚区，加快构建以现代工业为支撑的产业体系。着力抓增量补短板。大力实施产业集聚区“晋级计划”，坚持承接产业转移与扶持做大现有企业相结合，加快发展高成长性产业，培育壮大战略性新兴产业，改造提升传统产业，进一步做大产业集聚区规模，以增量带存量、调结构、补短板，增强产业集聚区的综合带动和支撑作用。今年全市产业集聚区主营业务收入力争超过1300亿元，其中，主营业务收入超过150亿元的产业集聚区2个，超过100亿元的5个。信阳高新技术产业开发区和潢川经济技术开发区要进一步扩大总量、提升水平、创出特色、当好示范，切实在创新升级上取得更大突破。着力抓集群建基地。大力实施“集群培育工程”，集中优势资源要素，支持高端制造、绿色食品、新型建材三大产业基地和区域性现代物流中心建设，不断培育壮大电子信息、绿色食品、新型建材、现代家居、现代物流、纺织服装、医药制造等主导产业集群。着力以智能手机、智能电视、智能影音为重点，打造智能终端产业链条；以主食产业化、畜禽深加工、茶叶深加工为重点，提升绿色食品优势产能；以提高新型保温建材产业技术水平和推广应用为重点，扩大新型建材产业规模；以加快承接家具、厨卫等产业链式转移为重点，做大现代家居产业；以物联网应用基础设施服务、集成服务、应用服务和电子商务等新兴产业为重点，发展现代物流业。大力支持产业集聚区培育百亿元特色产业集群，逐步将其培育成战略支撑产业。着力抓项目强支撑。继续开展产业集聚区“投资促进计划”和“重点项目攻坚计划”，力争产业集聚区完成投资900亿元。进一步提升高成长性和战略性产业比重，重点实施200个投资千万元以上的重大结构升级项目，完成投资200亿元以上；新开工重点攻坚项目50个，完成投资100亿元以上。加快推进信阳国际家居产业小镇、伯皇IT产业园、深圳手机产业园等重大项目建设，努力在集群招商和项目落地上取得更大成效。着力抓配套优环境。大力实施产业集聚区“功能提升工程”、“服务提质工程”和“创新驱动工程”，坚持建设服务与生产服务、项目配套与生活配套、发展保障与社会保障并重，建立更加有利于主导产业集群发展的体制机制。进一步完善基础设施配套，建立产业集聚区水、电、气稳定供应机制，确保建成区道路和水电管网全覆盖；加强公共服务平台建设，高标准规划建设一批产品展示中心和专业性批发交易市场，争取建设1—2个国家级质检中心和5—8个大型综合服务中心，实施2—3个省级重大科技专项。

　　（二）科学推进新型城镇化，着力在促进产城融合上下功夫。以加快建设鄂豫皖三省交界区域性中心城市为目标，坚持依城促产、以产兴城、产城融合，努力走出一条符合信阳实际的新型城镇化道路。坚持规划引领、优化布局。加快完成信阳市城市总体规划（2013-2030）修编工作，积极推进中心城区规划集中统一管理。统筹推进中心城市、县城和中心镇协调发展，加快推进中心城市“6+3”组团发展、新老城区互动发展、产业集聚区和城市融合发展。继续支持固始县加快发展。着力围绕建设一个产业集聚区、培育一个百亿元的特色主导产业、打造一个20万以上城市人口的“三个一”目标，支持潢川县加快发展成为区域性副中心城市，支持其他县城做大做强，把明港镇发展成为具有一定规模的城市。坚持以县为单位，按照产业、村庄、土地、生态环境和公共设施“五规合一”的要求，有序推进新农村建设，努力打造一批美丽乡村。坚持以人为本、有序转移。坚持产业为基、就业为本，强化住房、教育牵动，按照自愿、分类、有序的原则，重点解决一批已进城就业定居的农民工落户和一批城中村居民成建制转户，加快促进一批农村富余劳动力向城镇转移。继续深化各项配套改革，着力促进城镇基本公共服务向常住人口全覆盖，确保农业转移人口“进得来”、“落得住”、“转得出”。坚持生态文明、科学推进。尊重自然、顺应自然、保护自然，少挖山、不填湖、禁毁林、多栽树，切实保护好山体、水系、植被、湿地和生物多样性等生态资源，促进山水林城融合发展。创新科学推进新型城镇化的体制机制，着力解决好人往哪里去、钱从哪里来、土地怎么用、生态怎么保、城市怎么建的问题，不断提高城市规划建设管理科学化、精细化水平，加快推进生产空间集约高效、生活空间宜居适度、生态空间山青水秀、地下空间科学利用的新型城镇化。坚持城乡统筹、一体发展。加强中心城市路网建设，着力打通一批断头路，改造提升一批背街小巷，优先发展城市公共交通，有效解决老城区交通拥堵问题。继续加快浉河三期治理、城市内河治理、雨污分流等工程建设，加强城市地下管网配套建设，加大城市环境卫生整治力度，增强城市综合承载能力。大力实施中心城区的棚户区、城中村、旧商业街改造提升，进一步改善市民居住环境。加大城乡统筹发展力度，完善城镇交通、供排水、污水处理、生态水系工程等基础设施建设，增加教育、医疗、文化、体育等公共产品供给能力，加快推进城镇基础设施和公共服务向农村延伸，促进公共服务一体化。

　　（三）深化农村改革试验，着力在推进农业现代化上下功夫。按照稳定政策、改革创新、持续发展的总要求，以保障国家粮食安全和促进农民增收为核心，全面落实强农惠农富农政策，进一步加大农村改革发展力度，构建新型农业经营体系，强化农业基础地位，提升现代农业发展水平。继续深化农村改革发展综合试验区建设。认真贯彻落实中央和省委农村工作会议精神，进一步发挥先行先试优势，努力在农村土地流转、金融创新、专业合作、社会保障、城乡统筹、现代农业产业化、农业机械化等方面实现更大作为。继续实施高标准粮田“百千万”建设工程。加快高标准粮田建设和中低产田改造，继续推进农业综合开发、土地整理和复垦开发，力争今年建成高标准粮田50万亩以上，确保粮食产量稳定增长。继续实施现代农业产业化集群培育工程。大力培育粮油、畜禽、水产、茶叶、花木、林果、中药材等农业产业化集群，努力打造一批农业标准化示范区和特色高效农产品生产基地，力争市级以上农业产业化龙头企业突破350家。启动实施都市生态农业发展工程。在中心城区和有条件的县城周边，规划建设一批兼具城市“菜篮子”、生态绿化、休闲观光等功能的现代农业园区，发展新型都市农业业态。着力构建新型农业经营体系。综合运用政策引导、项目扶持、金融支持、技术服务等手段，进一步完善科技服务、农产品质量检测、动植物防疫、农村金融、农业保险等农业服务体系，加快培育发展专业大户、家庭农场、农民专业合作社、龙头企业等新型农业经营主体。支持符合条件的农民专业合作社承担实施财政支农建设项目，鼓励和引导工商资本到农村发展适合企业经营的现代种养业，着力构建农业产业化各类主体互利共赢的新型农业经营组织体系。

　　（四）积极培育新的增长点，着力在做大现代服务业上下功夫。大力培育服务业发展主导力量，不断促进现代服务业发展提速、比重提高、水平提升。做大做强高成长性服务业。以构建区域性现代物流枢纽为目标，加快推进金牛物流产业集聚区、商务中心区、特色商业区等载体基础设施建设，大力推进信阳国际家居产业小镇、信阳国际茶城、信阳义乌国际商贸城等重点项目建设，建立健全物流配送中心、冷链运输、冷链储藏等配套体系，完善邮政综合服务平台，培育大型物流企业，增强城市商业服务功能，力争在一些关键领域形成以信阳为中心或重要节点的现代物流网络。围绕构建区域性文化旅游中心，着力在资源融合、产业升级、市场开发、精品线路打造和公共服务提升上下功夫，加快推进大别山红色生态旅游一体化发展，加快国家5A级景区和国家旅游休闲度假区创建工作，尽快把信阳建成中国山水旅游休闲度假目的地。培育壮大新兴服务业。大力支持科技研发、工业设计、教育培训、商务服务、健康服务、会展服务、养老及家庭服务等新兴服务业发展，加快职业教育培训基地、区域性会展中心、健康服务产业基地、专业养老机构、社区服务中心建设，逐步将这些产业培育成服务业发展生力军。改造提升传统服务业。积极引入现代管理理念和新型商业模式，支持电商服务平台建设，鼓励大型商贸企业建设网上商城和中小微企业开设网店。创新经营模式，发展一批社区平价超市。大力发展城市综合体、文化旅游地产、养老地产等新兴业态，促进房地产业持续健康发展，满足居民多层次住房需求。

　　（五）强化支撑能力建设，着力在创造发展新优势上下功夫。继续加大投资力度，强化支撑体系建设，不断创造发展新优势。强化基础设施支撑。加快推进淮滨至豫皖界高速公路、息县杨店至邢集高速公路、淮河淮滨至息县航运基础设施、淮河公铁水一体化物流港等项目前期工作，实施8条国省干线公路改扩建，新建淮滨淮河二桥，争取开工建设明港机场，进一步提升信阳综合交通枢纽地位。以提高水资源保障和防汛抗旱减灾能力为重点，大力推进小型病险水库和大中型病险水闸除险加固、中小河流治理、大中型灌区续建配套与节水改造、淮干滩区居民和水库移民迁建、小农水重点县、农村饮水安全、水土保持等水利工程建设，争取开工建设出山店水库；优化能源结构和布局，推进西气东输二线（南阳—信阳）天然气管道项目和中石化（驻马店—信阳）成品油输油管道项目后续配套工程建设，积极实施坚强电网和新一轮农村电网改造升级工程，确保信阳东500千伏输变电工程上半年并网运行。强化科技创新支撑。深化科技体制机制创新，着力构建以企业为主体、市场为导向、政产学研相结合的自主创新体系。强化重大科技专项引领，加快科技成果转化，组织实施好豫南稻区籼改粳技术和豫南茶产业升级关键技术研发及产业化。加强科技创新平台建设，支持高等院校与龙头企业战略合作，支持龙头企业牵头创建国家级技术研究中心和产业技术创新战略联盟，支持新技术、新工艺、新产品研发，努力突破一批产业关键核心技术，增强产业核心竞争力。大力实施质量立市战略，深入开展政府质量奖和品牌创建活动，努力培育一批拥有自主知识产权和“信阳制造”的品牌企业。强化人力资源支撑。深入实施人才强市战略，着力创造一个有利于吸引人才、培养人才、留住人才的环境。大力支持具有本地特色的高等学校、职业院校和实训基地建设，努力培养一批科技实用人才。加快人力资源网上“超市”建设，着力在政府、企业和社会之间搭建一个招商与招工、培训与就业有效对接的服务平台，为我市更好地承接产业转移提供人力资源支持。强化信息化支撑。深化与三大通信集团的战略合作，加快智慧城市、无线城市、光网城市等工程建设。统筹推进城乡宽带网络升级改造，完成信阳移动4G网络升级改造项目。大力发展电子政务，推动民生和社会领域信息化应用。

　　（六）全面深化改革创新，着力在增强发展内生动力上下功夫。按照中央和省里统一部署，重点加快一批看得准、有条件、有权限的改革，努力在重要领域和关键环节取得实质进展和最大综合效益。深化重点领域改革。扎实推进政府职能转变，加快事业单位分类改革，按照简政放权、能放尽放的原则，继续清理、取消和下放一批行政审批服务事项。对保留的审批服务事项实行目录管理和流程再造，简化手续、优化流程，该放的放开放活，该管的管住管好。深入推进公共资源交易市场化改革，落实统一的市场准入制度，探索试行负面清单管理模式。改革工商登记制度，大力推进工商注册制度便利化，探索试行企业注册“一表通”，完善项目联审联批等制度，逐步由先证后照改为先照后证。推进预算编制改革和非税收入收缴改革，深化国库集中收付与公务卡改革，扩大政府采购范围和规模，防范和化解债务风险。加大政府向社会购买公共服务力度。继续深化户籍、林权、水利、供销和各项社会事业体制改革，稳妥推进资源型产品价格改革及基本民生和社会保障制度改革，基本完成农电体制改革。激发市场主体活力。继续深化国有企业改革，加快国有粮食企业产权制度改革和战略重组，完成市直机关所属企业脱钩改制工作，完善企业法人治理结构和内部管理制度，推进厂办大集体改革。以管资产为主进一步加强国有资产管理。认真落实促进非公经济发展的政策措施，研究制定非公企业进入特许经营领域的具体办法，鼓励非公企业参与国有企业改革，大力支持非公经济、混合型经济发展，切实为包括民营经济在内的各类市场主体创造公平、透明、可预期的市场环境，营造各种所有制经济依法平等使用生产要素、公平参与市场竞争、同等受到法律保护的体制环境。创新要素保障机制。全面规范推进人地挂钩政策试点工作，探索实行租让结合、分阶段出让的工业用地供应制度。科学配置用地计划指标，优先保证产业集聚区发展、民生建设和重大基础设施建设用地需求。加快农村集体建设用地和宅基地确权登记颁证工作，探索宅基地有偿退出机制。继续深化中心城区“三违”整治工作，规范土地管理秩序。进一步加强金融生态环境建设，大力发展普惠金融。充分发挥政府投融资平台作用，利用多层次资本市场、多种金融产品，支持企业上市融资，推动城投债、中小企业私募债、区域集优债等发债工作，扩大融资规模。繁荣金融主体，争取更多金融机构在信阳设立分支机构，支持在重点领域发起设立创业投资基金和产业投资基金。支持信阳银行参与中原银行筹建。积极探索农村金融改革创新试点，推动符合条件的农联社组建为农商行，推进村镇银行分支机构向乡镇延伸，支持具备条件的民间资本依法发起设立民营银行。优化小微企业融资环境，坚决打击非法集资，防范和化解金融风险。

　　（七）持续扩大对外开放，着力在提升开放型经济水平上下功夫。坚持以开放促改革、促转型、促发展，努力创造开放新优势。拓宽开放领域。抓住国家放宽外商投资市场准入机遇，加快服务业、农业、基础设施和社会事业等领域开放，紧盯大型跨国公司和知名企业，建立重点客商跟踪制度，力争引进一批具有战略支撑的重大项目。完善开放平台。大力支持和培育有条件的产业集聚区创新升级，加快完善海关、口岸、物流园区建设，全方位打造开放平台。继续加强与央企、省企的战略合作，积极争取境内外战略投资者在信阳设立地区总部和区域性的研发、物流、销售、结算、后台服务中心。着力办好第22届信阳国际茶文化节，力争在招商引资上取得更大成效。全力办好“中国光彩事业信阳行”活动，深度谋划，精准对接，力争签约和落户一批重大项目。提升开放成效。更加有针对性地围绕产业集聚区发展规划和主导产业链条图谱，分行业制定龙头企业和产业集群引进行动计划，组织专业精干力量，深化“四一”招商行动，以龙头企业或重大项目为产业链内核，加快引进缺失链条、补强薄弱链条、提升关键链条，努力形成产业集群引进和企业抱团转移的倍增效应。更加有针对性地发挥企业在招商中的主体作用、商会协会的桥梁作用和统战系统及工商联系统的依托作用，大力开展以商招商和中介招商，努力引进一批高端和高成长性项目。更加有针对性地强化项目落地，完善招商项目跟踪服务、择地落户和评价奖惩机制，提高合同履约率、项目开工率、资金到位率和投入产出率，确保更多项目在信阳扎根开花结果。

　　（八）加快美丽信阳建设，着力在推动可持续发展上下功夫。坚持把生态文明理念贯穿于经济社会发展全过程，以更大的决心和更有力的举措，推进绿色循环低碳发展，保护生态环境，打造天蓝、地绿、水净的美丽信阳。加强生态系统建设。继续加强淮河源国家重点生态功能区保护，深入开展全国绿化模范城市创建工作，大力实施林业生态市建设提升工程，加强退耕还林、重点地区防护林、生态廊道等工程建设，构建绿色生态体系，提升生态自然修复能力。全年完成造林33.8万亩、森林抚育和改造35.5万亩。加强环境综合治理。大力实施“蓝天工程”、“碧水工程”、“乡村清洁工程”，加强PM2.5监测治理，继续推进南湾湖国家重点湖泊生态保护和环境治理，加强农村面源污染治理和土地污染治理，进一步改善和提高环境质量。加强节能减排工作。实行更加严格的环境准入制度和固定资产投资节能评估审查制度，严禁引进高污染、高耗能项目。发挥公共机构和企业的引领示范作用，增强全社会节能减排内生动力。强化环境容量控制，建立项目环评审批与当地容量挂钩机制，完善污染物排放许可证制度。全面推进节能降耗行动计划，大力实施一批循环经济、清洁生产和节能降耗示范工程，加快新型保温建材开发应用，推进资源节约集约利用，提升循环经济发展水平。构建生态文明长效机制。完善绩效考核办法，健全生态补偿机制，实行最严格的源头保护、损害赔偿和责任追究制度。倡导绿色生活方式和消费理念，引导公众积极参与生态文明建设。

　　（九）提升公共服务水平，着力在保障和改善民生上下功夫。继续加大对民生领域投入力度，认真落实促进城乡居民增收的政策措施，进一步提高基本公共服务均等化程度，努力让人民群众过上更好生活。更加重视就业创业。继续把就业作为经济社会发展的优先目标，实施更加积极的就业政策，发挥创业带动就业的倍增效应，做好高校毕业生、农村转移劳动力、失地农民、下岗失业人员的就业工作，加大困难就业群体帮扶力度，始终保持零就业家庭动态归零。继续实施全民技能振兴工程、职教攻坚工程、技能人才回归工程，完成各类职业技能培训20万人次，推动实现更高质量就业。加快基层人力资源社会保障服务平台建设，力争实现全覆盖。积极构建和谐劳动关系，着力解决拖欠农民工工次问题，维护劳动者合法权益。更加重视社会保障。坚持保基本、兜底线、促公平、可持续，进一步完善各项社会保险和社会救助制度，扩大社会保障覆盖范围，提升社会保险统筹层次，提高企业退休人员基本养老金、城乡居民低保和农村五保对象供养标准。进一步扩大住房公积金制度覆盖面，更好地发挥住房公积金制度在住房保障中的重要作用。加大保障性安居工程建设，争取新开工保障性住房17749套，基本建成15532套。更加重视社会事业发展。加大对基础教育投入力度，完善基本公共教育服务体系，优化城乡教育资源布局，制定中心城区教育专项规划，加强教师队伍建设，加快普及学前教育，推进义务教育均衡发展和普通高中、职业教育多样化发展，支持高等教育事业发展，加快大别山干部学院建设。深化医药卫生体制改革，抓好县级公立医院综合改革试点，巩固完善基本药物制度和基层医疗卫生机构运行新机制，探索乡村一体化管理新模式。完善基层医疗卫生服务体系，加强基层医疗基础设施建设，促进优质医疗资源纵向流动。抓好重大传染病防控救治工作。积极发展中医药事业。创新文化体制改革，推动政府部门由办文化向管文化转变，推进国有经营性文化单位转企改制，抓好公益性文化事业单位内部机制改革，支持文化产业规模化、专业化、品牌化发展。大力实施文化惠民工程，加快广播影视、新闻出版事业发展，繁荣文学艺术创作，加强网络文化建设，加大文化市场管理，持续开展“扫黄打非”，进一步丰富群众文化生活。加强重点文物保护单位和重要革命旧址抢救修复。深入开展全民健身运动，积极构建基本公共体育服务体系，促进群众体育和竞技体育全面发展。坚持计划生育基本国策，进一步加强人口和计划生育工作。积极应对人口老龄化，完善社会养老服务体系，发展养老事业。大力发展慈善、社会福利、妇女儿童、残疾人等事业。关爱留守儿童、留守妇女和留守老人。更加重视扶贫开发。深入推进大别山集中连片特殊困难地区扶贫攻坚，全市新建改建农村公路400公里、桥梁3000延米，解决50万农村居民及师生饮水安全问题，再实现11万农村贫困人口稳定脱贫。更加重视安全和安全生产。强化食品药品安全监管责任，用严格的标准、严密的监管、严厉的惩处、严肃的问责，确保人民群众“舌尖上的安全”。严格落实“党政同责、一岗双责、齐抓共管”安全生产责任制，强化公共安全体系和企业安全生产基础建设，深化重点行业和领域整治，严厉打击非法违法和违规违章生产行为，加强校园安全建设，坚决消除安全隐患，坚决遏制重特大事故发生。更加重视社会治理创新。深化平安信阳建设，加强法制宣传教育，统筹实施市应急指挥中心、智能交通和治安管理视频监控系统建设，构建全覆盖的社会治安防控体系，严厉打击违法犯罪行为。加强社区和基层综合服务管理平台建设，完善和创新流动人口、特殊人群管理服务机制，有效排查化解社会矛盾。进一步加大力度，解决好拆迁安置遗留问题。加强和改进信访工作，集中处置一批信访积案，依法按政策解决群众的合理诉求，维护社会大局和谐稳定。

　　继续加强新形势下国防教育，增强全民国防意识，抓好民兵预备役和人民防空工作，积极支持国防建设和驻信解放军、武警部队、消防部队建设；认真落实新时期支持国防和军队建设、社会化拥军优属的政策措施，继续以军地互办实事为抓手，深入开展“双拥”共建，巩固和发展军政军民团结。大力支持工会、共青团、妇联和科协、文联、社科联、工商联、红十字会等人民团体发挥桥梁纽带作用，全面落实民族、宗教政策，切实做好外事、侨务、对台工作。继续抓好机关事务、地质、气象、地震、档案、史志等各项工作。认真做好第三次全国经济普查。

　　今年，在完成省定“十项重点民生工程”的同时，继续抓好“十件实事”，具体安排意见已经印发，请予审议。

　　四、以改革创新精神加强政府自身建设

　　深入开展党的群众路线教育实践活动，扎实开展“服务质量提升年”活动，深入推进“两转两提”，持续提高政府工作科学化、法治化水平，树立政府务实为民清廉形象。

　　（一）加快建设服务型政府。围绕市场在资源配置中起决定作用和更好发挥政府作用，进一步理顺政府与企业、市场、社会的关系，强化政府公共服务、市场监管、社会管理、环境保护等职责，推动政府职能加快向创造良好发展环境、提供优质公共服务、维护社会公平正义转变。进一步提高政府公共服务效能，推动公共资源更多地向民生领域倾斜，努力为人民群众提供更好、更优、更均衡的公共产品服务。加强政务诚信建设，以政务诚信带动商务诚信和社会诚信，提高政府公信力和执行力。严控机构编制，确保财政供养人员只减不增。

　　（二）加快建设法治型政府。坚持依法行政，严格按照法定权限和程序行使权力、履行职责，坚持用制度管权管事管人，推动政府工作法治化。坚决执行人大及其常委会决议决定，自觉接受法律和工作监督，支持人大代表依法行使职权。支持政协参政议政，主动接受政协民主监督。认真办理人大代表建议和政协委员提案，广泛听取民主党派、工商联、无党派人士和人民团体意见。坚持从严治政，加强行政监察、执法监督和审计监督，完善行政复议制度，强化行政执法责任和过错追究，规范行政行为。坚持依法科学民主决策，完善公众参与、专家论证、风险评估、合法性审查和集体讨论决定等决策程序，提高决策透明度和公众参与度。深化政务公开，加强舆论和社会监督，让行政权力在阳光下运行，让政府更好地服务人民，让人民更好地监督政府。

　　（三）加快建设创新型政府。深化学习型机关建设，加强教育培训，增强公务员队伍的创新意识、大局意识、责任意识，不断开创各项工作新局面。积极创新工作机制，大力倡导务实重干，强化绩效评估，树立正确考核导向，努力把各方面精力引导到转变发展方式、提高质量效益上来，多做打基础、增后劲、管长远的工作，不搞“形象工程”、“政绩工程”，努力创造经得起实践、人民和历史检验的业绩。

　　（四）加快建设廉洁型政府。严格落实中央“八项规定”和省市关于加强作风建设的各项规定，突出解决“四风”问题，切实改进工作作风。坚持廉洁从政，严格落实廉政建设责任制，坚决反对和惩治腐败，坚决查处损害群众利益的行为，以反腐败斗争实效保障发展、取信于民。坚持从严从俭，严守规矩，刚性约束，严格控制一般性支出，确保“三公”经费只减不增。坚决禁止新建楼堂馆所，坚决禁止舌尖上的浪费、车轮上的铺张和节日期间的不正之风，切实把有限财力投入到加快发展、改善民生上，使发展成果更多更公平地惠及人民群众。

　　各位代表！

　　新的一年面临新的机遇，新的一年充满新的希望。让我们高举中国特色社会主义伟大旗帜，紧密团结在以习近平同志为总书记的党中央周围，肩负起全市人民的重托，凝聚起全市人民的智慧力量，持续求进，务实发展，积极作为，确保完成全年经济社会发展目标，为勇当中原经济区建设前锋、全面提升魅力信阳建设水平、持续改善人民群众生活而努力奋斗！
